# Cell death of alveolar lymphocytes and monocytes is negatively correlated with driving pressure and mechanical power in patients with acute respiratory distress syndrome

**DOI:** 10.1186/s40001-023-01607-4

**Published:** 2024-01-03

**Authors:** Shaw-Woei Leu, Chien-Min Chu, Chia-Jung Chung, Chih-Yu Huang, Chao-Hung Wang, Li-Fu Li, Huang-Pin Wu

**Affiliations:** 1 Department of Thoracic Medicine, Chang Gung Memorial Hospital, Linkou, 33305 Taiwan; 2 College of Medicine, Chang Gung University, Taoyuan, 33302 Taiwan; 3 Division of Pulmonary, Critical Care and Sleep Medicine, Chang Gung Memorial Hospital, Keelung. 222, Maijin Rd., Anle Chiu, Keelung, 20401 Taiwan; 4 Heart Failure Research Center, Division of Cardiology, Chang Gung Memorial Hospital, Keelung, 20401 Taiwan

**Keywords:** Bronchoalveolar lavage fluid cells, Cell death, Acute respiratory distress syndrome, Lung compliance, Driving pressure, Mechanical power

## Abstract

**Background:**

Pathogenesis of acute respiratory distress syndrome (ARDS) involves immune cell death and removal from the injured lungs. ARDS severity is related to lung compliance. However, the correlation between the respiratory mechanics and alveolar immune cell death in patients with ARDS remains unclear.

**Methods:**

Twenty-four patients with respiratory failure and ARDS were enrolled in the intensive care unit between November 2019 and November 2021. Neutrophil extracellular traps (NETs) and cell death of lymphocytes and monocytes in bronchoalveolar lavage fluid were detected on days 1 and 8.

**Results:**

Lung compliance was positively correlated with the cell death percentage of alveolar CD4/CD8 lymphocytes and monocytes on day 8 (Pearson’s correlation coefficient (*r*) = 0.554, *p* = 0.005; *r* = 0.422, *p* = 0.040; *r* = 0.569, *p* = 0.004, respectively). There was no association between lung compliance and the percentage of alveolar NETs on days 1 and 8. The cell death percentages of alveolar CD4/CD8 lymphocytes and monocytes were negatively correlated with driving pressure (DP) on days 1 (*r* = − 0.440, *p* = 0.032; *r* = − 0.613, *p* = 0.001; *r* = -0.557, *p* = 0.005, respectively) and 8 (*r* = − 0.459, *p* = 0.024; *r* = − 0.407, *p* = 0.048; *r* = − 0.607, *p* = 0.002, respectively). The cell death percentages of alveolar CD4/CD8 lymphocytes and monocytes were also negatively correlated with mechanical power (MP) on days 1 (*r* = − 0.558, *p* = 0.005; *r* = − 0.593, *p* = 0.002; *r* = − 0.571, *p* = 0.004, respectively) and 8 (*r* = − 0.539, *p* = 0.007; *r* = − 0.338, *p* = 0.107; *r* = − 0.649, *p* < 0.001, respectively). The percentage of alveolar NETs on days 1 and 8 was not associated with DP or MP.

**Conclusion:**

Patients with higher cell death rates of alveolar CD4/CD8 lymphocytes and monocytes exhibited lower DP and MP. Patients with less cell death of alveolar CD4/CD8 lymphocytes and monocytes required more DP or MP to maintain adequate ventilation.

**Supplementary Information:**

The online version contains supplementary material available at 10.1186/s40001-023-01607-4.

## Background

The pathophysiology of acute respiratory distress syndrome (ARDS) is characterized by inflammation-mediated disruption of alveolar-capillary permeability, edema formation, reduced alveolar clearance and de-recruitment, decreased compliance, increased pulmonary vascular resistance, and gas exchange abnormalities due to shunting and ventilation–perfusion mismatch [1]. Despite its high mortality rate, no proven medicines can reduce its mortality [2].

Lung injury in the exudative phase begins with the activation of alveolar macrophages by microbial or cellular injury products. Cytokines and chemokines released by macrophages and lymphocytes recruit and activate circulating neutrophils. Activated neutrophils contribute to injury by releasing mediators [2]. For example, tumor necrosis factor induces the expression of tissue factor, leading to platelet aggregation, microthrombus formation, as well as intra-alveolar coagulation and the development of hyaline membrane structures. Although they assist in killing pathogens, they also injure the normally tight alveolar endothelial–epithelial barrier. In the proliferative phase, the immune response is recalibrated by clearance of pathogens and damaged host cells from alveolar space. This involves immune cell death and removal, expansion of resident fibroblasts, interstitial matrix reformation, and regrowth of the alveolar epithelium by the differentiation of type II alveolar cells into type I cells [1].

The formation of neutrophil extracellular traps (NETs) is increased in addition to alveolar immune cell death in ARDS [3]. NETs are formed by dying neutrophils that release DNA, histones and granular proteins, including myeloperoxidase and neutrophil elastase [4]. NETs are essential for defense against pathogen invasion, and their increased release or degradation leads to a higher risk of poor infection control. NETs also contain several pro-inflammatory and cytotoxic molecules that can exacerbate inflammation and lung tissue injury. NETs have been detected in the lungs, where they are involved in ARDS development [5, 6]. Furthermore, NETs could induce macrophage pyroptosis [7] and can suppress CD4 lymphocyte responses through metabolic and functional exhaustion [8].

The best strategy for reducing ARDS mortality is to avoid ventilator-induced lung injury (VILI), which is lung-protective ventilation [9]. It is potentially helpful to approach the driving pressure (DP) and mechanical power (MP) for the ventilatory management of ARDS [9–11]. Thus, we hypothesized that NETs and death of lymphocytes and monocytes in the lungs of patients with ARDS might be associated with respiratory mechanics. It is critical to understand the pathogenesis and complex interactions between immune cell death and respiratory mechanics in ARDS to identify potential therapeutic targets. We designed a cohort study to determine the relationship between alveolar immune cell death and respiratory mechanics in patients with ARDS.

## Methods

### Participants and definitions

According to our hospital regulations, respiratory samples from patients with suspicious coronavirus disease 2019 (COVID-19) cannot be used for experiments in the COVID-19 pandemic. From November 2019 to November 2021, only 31 patients with severe pneumonia, respiratory failure, and ARDS who were admitted to the intensive care unit (ICU) were enrolled into this study. The exclusion criteria included COVID-19 pneumonia, absence of invasive ventilator support, or death before bronchoalveolar lavage (BAL). This study was approved by the Institutional Review Board of Chang Gung Memorial Hospital (201601731A3, 201601731A3C501, and 201801835A3). The protocol was performed in accordance with the guidelines and regulations of the Declaration of Helsinki. The patients’ close family members provided informed consent for the collection of samples and subsequent analysis. Patients who survived for greater than 28 days after ICU admission were defined as survivors. All comorbidities and patient history were recorded.

ARDS was defined according to the Berlin definition [12]. Pneumonia was defined as a new abnormal infiltration on chest radiography with respiratory symptoms or fever. Sepsis and septic shock were defined according to Sepsis-3 guidelines [13]. Respiratory failure was defined as a ventilatory dysfunction requiring invasive ventilator support. Acute kidney injury was defined according to the stage 1, 2, or 3 in the Kidney Disease Improving Global Outcomes guidelines [14]. Jaundice was defined as total bilirubin > 2 mg/dL, whereas thrombocytopenia was defined as a platelet count < 150,000/μL. Disease severity was assessed based on the Acute Physiology and Chronic Health Evaluation II score [15].

Standard treatment was provided to all patients according to the guidelines [16], including initial fluid resuscitation, antibiotics use, vasopressor to maintain blood pressure, sedation with or without neuromuscular blocking agents, renal replacement therapy for acute renal failure, and low tidal ventilation, according to the condition of the patient. The target of sedation depth was set on–4 (open eyes or move to physical stimulation) of Richmond Agitation Sedation Scale.

### Ventilator settings

Patients routinely received pressure-controlled ventilation (PCV). Following intubation, all patients were routinely ventilated with a target tidal volume of approximately 6–8 mL/kg predicted body weight (PBW) and minimal pressure control level after adjusting inspiratory time. The goal was to maintain an inspiratory plateau pressure of less than 30 cm H_2_O. The positive end-expiratory pressure (PEEP) level and fraction of inspired oxygen were adjusted to maintain an arterial partial pressure of oxygen of > 60 mmHg or oxygen saturation by pulse oximetry of > 90% according to the suggestion of the ARDS network [17]. The ventilator settings were adjusted 2 h after the first setting. Ventilator weaning and adjustments were performed at regular intervals (every 8 h) and as necessary, based on the general weaning guidelines and clinical practice of our respiratory therapy department [18].

### Measurement of respiratory mechanics

Respiratory mechanics were measured under PCV or transiently under PCV if patients were under weaning mode, such as synchronized intermittent mandatory ventilation with pressure support ventilation (PSV) or PSV. Compliance (C), DP, and MP for pressure-targeted ventilation were calculated per 8 h/day according to the equations, using respiratory rate (RR), tidal volume size (L) (V_T_), inspiratory airway pressure (cm H_2_O) (P_insp_), and positive end-expiratory pressure (cm H_2_O) (PEEP) [10, 19–21]:

$${\text{C }}\left( {{\text{mL}}/{\text{cm H}}_{{2}} {\text{O}}} \right) \, = { 1}000 \, \times {\text{ V}}_{{\text{T}}} / \, ({\text{P}}_{{{\text{insp}}}} - {\text{PEEP}})$$

$${\text{DP }}\left( {{\text{cm H}}_{{2}} {\text{O}}} \right) \, = {\text{ P}}_{{{\text{insp}}}} - {\text{PEEP}}$$

$${\text{MP }}\left( {{\text{J}}/{\text{min}}} \right) \, = \, 0.0{98 } \times {\text{ RR }} \times {\text{ V}}_{{\text{T}}} \times \, \left( {{\text{DP }} + {\text{ PEEP}}} \right)$$

### Preparation of BAL mononuclear cells and neutrophils

BAL was performed in the lung segment showing consolidation and infiltration on chest radiography, which was suspected as an area of pneumonia and ARDS within 48 h of admission to the ICU (at 08:30 AM). For the BAL fluid, the first aliquot of sterile saline (60 mL) was instilled via a bronchoscope wedged into the segmental bronchus. The instilled saline was gently retrieved using negative-suction pressure of < 100 mmHg. Negative-suction pressure should be adjusted to avoid visible airway collapse. Two aliquots of sterile saline (60 mL) were prepared in the same manner as the first aliquot. A BAL fluid was regarded representative while it fulfilled two criteria: (i) volume > 40 ml, (ii) total cell count > 100 cells/µl. To remove debris, the recovered volume was filtered through a cotton gauze and stored in heparin tubes (5000 U/50 mL recovered volume). The day of the first BAL was defined as day 1. Additional BAL was performed on day 8, unless the patient died or was discharged from the ICU. BAL mononuclear cells and neutrophils were isolated via differential density gradient centrifugation within 2 h of collection after red blood cell lysis over Ficoll-Plaque (Amersham Biosciences, Amersham, UK).

### Flow cytometric analysis of BAL mononuclear cells

BAL mononuclear cells (5 × 10^5^) were suspended in 50 μL of phosphate-buffered saline and incubated in the dark for 15 min at room temperature with 10 μL of annexin-V_PE_, CD4_ECD_, 7-aminoactinomycin D (7-AAD), CD11b_PC7_, CD8_APC_, CD3_Alexa Fluor 700_, and CD14_APC-750_ antibodies. Death of CD4/CD8 lymphocytes and monocytes was detected with positive 7-AAD and annexin-V staining using an eight-color flow cytofluorometer (Beckman Coulter, Brea, CA) (Additional file 1: Fig S1).

### Microscopic imaging of NETs

A total of 5 × 10^5^ neutrophils were seeded in a well of a Corning BioCoat Collagen I 96-wells plate (Thermo Fisher Scientific Inc, Waltham, MA). Neutrophils were incubated with 16 μM Hoechst 33342 (Thermo Fisher Scientific Inc, Waltham, MA) for 10 min and washed twice with RPMI 1640. The neutrophils were incubated with 167 nM Sytox Green (Thermo Fisher Scientific Inc, Waltham, MA) for 30 min and washed twice with RPMI 1640. NETs images were recorded using a Nikon Ti-E imaging system with a 20 × objective (Nikon, Tokyo, Japan). A set of images (Exc/Em:350/461 nm (Hoechst) and 504/523 nm (Sytox Green)) was taken with a CoolSNAP MYO CCD camera (Teledyne Photometrics, Tucson, AZ) and four fields of view (each 450 × 340 μm) were captured (Additional file 1: Fig S2).

To quantify NETs, images were processed using Fiji software (National Institutes of Health, Bethesda, MD). Hoechst staining was used to determine the total number of neutrophils. The number of Sytox Green-positive neutrophils (35–68 μm^2^) or NETs (> 68 μm^2^) was divided by the total number of neutrophils. The NETs level was defined as the percentage of NETs to the total neutrophil number.

### Statistical analyses

Statistical analyses were performed using SPSS Statistics, version 27.0.1 for Mac (IBM Inc., Armonk, NY, USA). Correlations between continuous variables were examined using the Pearson’s correlation test to obtain the Pearson correlation coefficient (*r*). Statistical significance was set at *p* < 0.05.

## Results

Table 1 shows the clinical characteristics of patients with ARDS. Initially, 31 patients were enrolled in this study; however, data of 3 patients were not used because of a lack of sufficient cells from BAL for flow cytometry. BAL was not performed on day 8 in 2 patients due to successful ventilator discontinuation, and 2 patients died within 7 days. Only 24 patients had complete BAL data on days 1 and 8 for analysis. Respiratory mechanics for serial 8 days and the percentages of alveolar cell death on days 1 and 8 are shown in Tables 2, 3, respectively.

**Table 1:** Clinical characteristics of patients with ARDS

	All patients (n = 24)
Age (years)	76.5 (59.8–84.5)
Male	17 (70.8)
APACHE II score on Day 1	26.0 (23.0–28.0)
SOFA score on Day 1	8.5 (6.0–10.8)
History	
Chronic obstructive pulmonary disease	5 (20.8)
Heart failure	1 (4.2)
Hypertension	11 (45.8)
Diabetes mellitus	9 (37.5)
Previous cerebral vascular accident	5 (20.8)
End stage renal disease	0 (0.0)
Liver cirrhosis	1 (4.2)
Active malignancy	1 (4.2)
Adverse event	
New arrhythmia	2 (8.3)
Gastrointestinal bleeding	5 (20.8)
Acute kidney injury	8 (33.3)
Shock	7 (29.2)
Thrombocytopenia	7 (29.2)
Jaundice	3 (12.5)
Bacteremia	4 (16.7)
PaO_2_/FiO_2_ ratio (mm Hg)	134.4 (107.6–191.5)
Mild, 200 < PaO_2_/FiO_2_ ≤ 300	5 (20.8)
Moderate, 100 < PaO_2_/FiO_2_ ≤ 200	16 (66.7)
Severe, PaO_2_/FiO_2_ ≤ 100	3 (12.5)
Management	
Glucocorticoid	7 (29.2)
Vasopressor	7 (29.2)
Prone ventilation	2 (8.3)
Extracorporeal membrane oxygenation	0 (0.0)
Pathogen detected in airway	
*Stenotrophomonas maltophilia*	2 (8.3)
*Influenza virus*	2 (8.3)
*Staphylococcus aureus*	1 (4.2)
*Acinetobacter baumannii*	1 (4.2)
*Klebsiella pneumoniae*	1 (4.2)
*Escherichia coli*	1 (4.2)
*Enterobacter species*	1 (4.2)
*Candida species*	1 (4.2)
*Streptococcus pneumoniae*	1 (4.2)
APACHE II score on Day 8	23.0 (17.3–27.0)
SOFA score on Day 8	5.5 (3.0–9.0)
28 day mortality	1 (4.2)

Data are presented as number (%) or median (25–75% interquartile range)

*ARDS* acute respiratory distress syndrome, *APACHE* Acute Physiology and Chronic Health Evaluation, *SOFA* Sequential Organ Failure Assessment, *PaO*_*2*_ partial pressure of oxygen, *FiO*_*2*_ fraction of inspired oxygen

**Table 2:** Serial respiratory mechanics in patients with acute respiratory distress syndrome

Dynamic lung compliance on Day 1, mL/cm H_2_O	29.799 (23.989–40.251)
Dynamic lung compliance on Day 2, mL/cm H_2_O	34.640 (25.753–39.603)
Dynamic lung compliance on Day 3, mL/cm H_2_O	36.510 (30.176–47.595)
Dynamic lung compliance on Day 4, mL/cm H_2_O	39.791 (30.422–50.338)
Dynamic lung compliance on Day 5, mL/cm H_2_O	36.835 (28.504–56.143)
Dynamic lung compliance on Day 6, mL/cm H_2_O	36.492 (27.967–50.611)
Dynamic lung compliance on Day 7, mL/cm H_2_O	36.781 (26.021–49.856)
Dynamic lung compliance on Day 8, mL/cm H_2_O	35.667 (24.006–53.004)
Mean dynamic lung compliance, mL/cm H_2_O	35.702 (29.375–49.536)
Tidal volume on Day 1, mL/kg PBW	9.300 (7.375–10.525)
Tidal volume on Day 2, mL/kg PBW	9.050 (7.650–10.225)
Tidal volume on Day 3, mL/kg PBW	8.650 (7.400–10.650)
Tidal volume on Day 4, mL/kg PBW	8.600 (7.625–9.975)
Tidal volume on Day 5, mL/kg PBW	8.950 (7.725–10.900)
Tidal volume on Day 6, mL/kg PBW	8.950 (7.825–10.900)
Tidal volume on Day 7, mL/kg PBW	8.900 (7.525–11.525)
Tidal volume on Day 8, mL/kg PBW	8.750 (7.200–9.500)
Mean tidal volume, mL/kg PBW	9.175 (7.769–10.125)
Driving pressure on Day 1, cm H_2_O	16.000 (14.000–19.500)
Driving pressure on Day 2, cm H_2_O	14.000 (12.000–17.500)
Driving pressure on Day 3, cm H_2_O	13.000 (10.500–15.000)
Driving pressure on Day 4, cm H_2_O	12.000 (10.000–15.000)
Driving pressure on Day 5, cm H_2_O	12.500 (10.000–15.750)
Driving pressure on Day 6, cm H_2_O	13.000 (10.000–17.750)
Driving pressure on Day 7, cm H_2_O	13.500 (10.000–20.000)
Driving pressure on Day 8, cm H_2_O	13.500 (10.000–18.750)
Mean driving pressure, cm H_2_O	14.250 (11.781–16.406)
Mechanical power on Day 1, J/min	29.314 (24.047–33.682)
Mechanical power on Day 2, J/min	22.734 (15.876–26.403)
Mechanical power on Day 3, J/min	20.435 (15.430–25.175)
Mechanical power on Day 4, J/min	17.097 (15.791–26.752)
Mechanical power on Day 5, J/min	19.677 (13.928–28.360)
Mechanical power on Day 6, J/min	18.891 (15.472–29.968)
Mechanical power on Day 7, J/min	22.813 (15.847–29.660)
Mechanical power on Day 8, J/min	21.227 (15.248–28.128)
Mean mechanical power, J/min	21.312 (19.190–27.773)

Data are median (25–75% interquartile range)

*PBW* predicted body weight

**Table 3:** Alveolar cell death in patients with acute respiratory distress syndrome

Alveolar cell death on Day 1, %	
CD4 lymphocytes	32.670 (17.773–69.075)
CD8 lymphocytes	45.095 (7.340–71.198)
Monocytes	4.355 (0.560–29.688)
Neutrophil extracellular traps	12.800 (8.150–18.200)
Alveolar cell death on Day 8, %	
CD4 lymphocytes	42.820 (30.178–61.265)
CD8 lymphocytes	40.505 (14.863–70.843)
Monocytes	21.465 (3.368–32.363)
Neutrophil extracellular traps	14.200 (7.550–21.525)

Data are median (25–75% interquartile range)

### Correlation between dynamic lung compliance and alveolar cell death

**Fig. 1:**
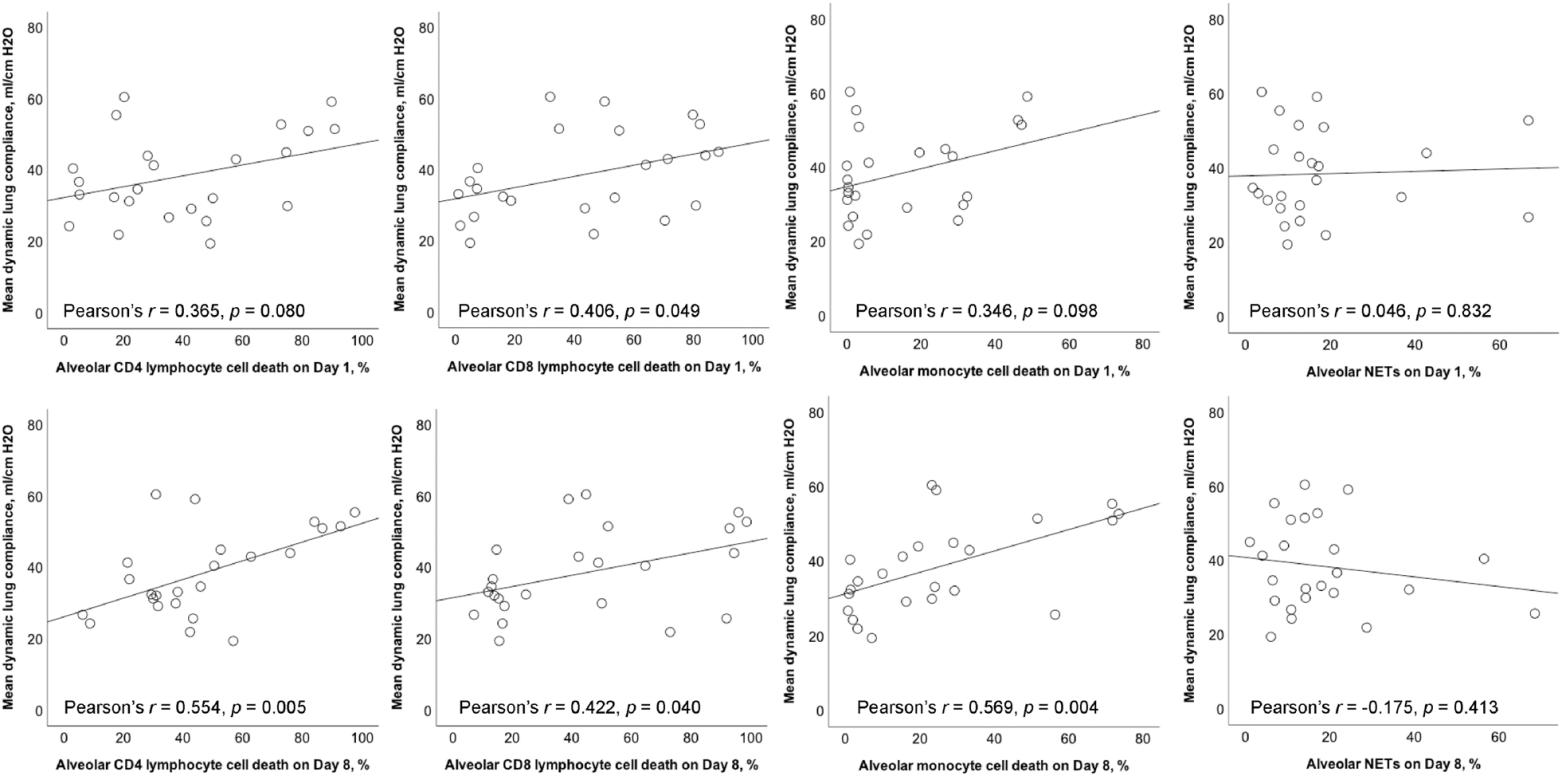
Scatterplots showing the Pearson correlation with linear mean regression lines between mean dynamic lung compliance and different alveolar cell death on Days 1 and 8. Pearson correlation coefficient (*r*) was calculated. *NETs* neutrophil extracellular traps

Alveolar CD8 lymphocyte death on day 1 was positively correlated with mean dynamic lung compliance (*r* = 0.406, *p* = 0.049) (Fig. 1). Alveolar CD4 lymphocyte and monocyte cell death on day 1 appeared to be positively associated with mean dynamic lung compliance, but the correlation was not significant. Mean dynamic lung compliance was positively correlated with alveolar CD4/CD8 lymphocyte and monocyte cell death on day 8 (*r* = 0.554, *p* = 0.005; *r* = 0.422, *p* = 0.040; *r* = 0.569, *p* = 0.004, respectively). There was no association between mean dynamic lung compliance and alveolar NETs on days 1 and 8.

### Correlation between tidal volume and alveolar cell death

Alveolar CD4/CD8 lymphocyte and monocyte cell death on day 1 appeared to be negatively associated with mean tidal volume, but the correlation was not significant (Additional file 1: Fig S3). There was no association between the mean tidal volume, alveolar CD4/CD8 lymphocyte death, and monocyte death on day 8. Mean tidal volume did not correlate with alveolar NETs on days 1 and 8.

### Correlation between DP and alveolar cell death

**Fig. 2:**
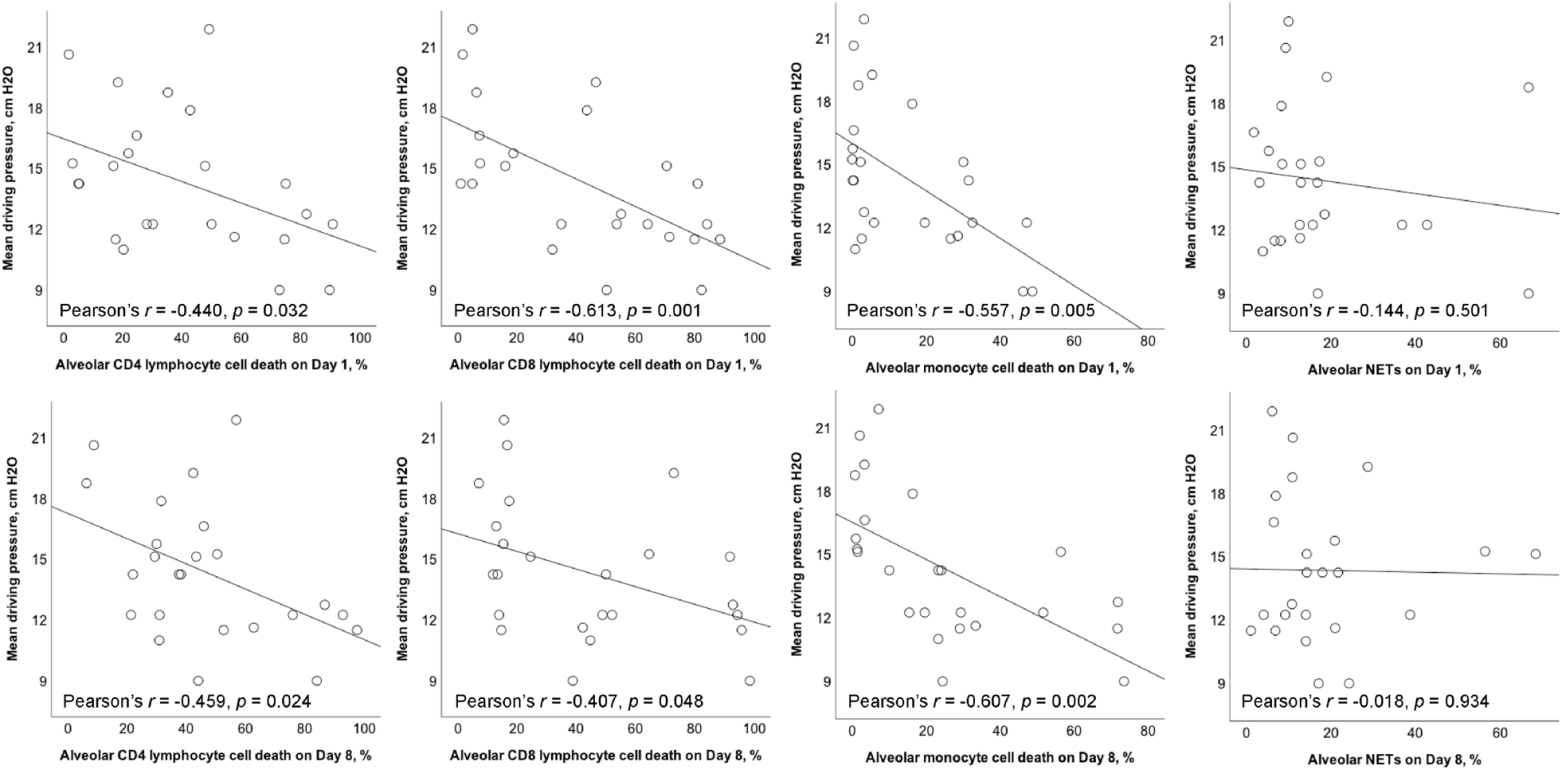
Scatterplots showing the Pearson correlation with linear mean regression lines between mean driving pressure and different alveolar cell death on Days 1 and 8. Pearson correlation coefficient (*r*) was calculated. *NETs* neutrophil extracellular traps

The mean DP was negatively correlated with alveolar CD4/CD8 lymphocyte and monocyte cell death on day 1 (*r* = − 0.440, *p* = 0.032; *r* = − 0.613, *p* = 0.001; *r* = − 0.557, *p* = 0.005, respectively) (Fig. 2). Mean DP was negatively correlated with alveolar CD4/CD8 lymphocyte and monocyte cell death on day 8 (*r* = − 0.459, *p* = 0.024; *r* = − 0.407, *p* = 0.048; *r* = − 0.607, *p* = 0.002, respectively). There was no association between mean DP and alveolar NETs on days 1 and 8.

### Correlation between MP and alveolar cell death

**Fig. 3:**
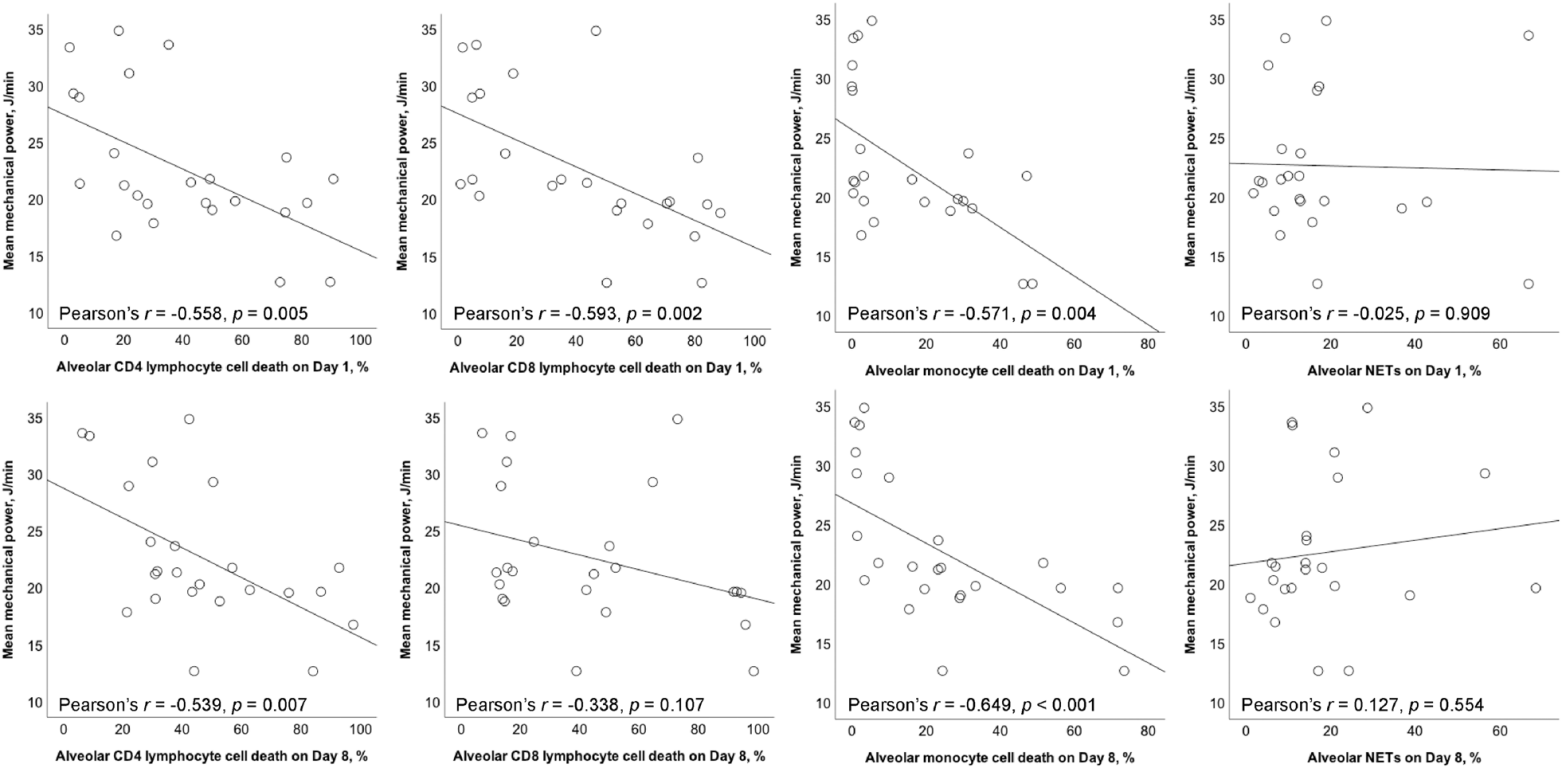
Scatterplots showing the Pearson correlation with linear mean regression lines between mean mechanical power and different alveolar cell death on Days 1 and 8. Pearson correlation coefficient (*r*) was calculated. *NETs* neutrophil extracellular traps

The mean MP was negatively correlated with alveolar CD4/CD8 lymphocyte and monocyte cell death on day 1 (*r* = − 0.558, *p* = 0.005; *r* = − 0.593, *p* = 0.002; *r* = − 0.571, *p* = 0.004, respectively) (Fig. 3). The mean MP was negatively correlated with alveolar CD4 lymphocyte and monocyte cell death on day 8 (*r* = − 0.539, *p* = 0.007; *r* = − 0.649, *p* < 0.001, respectively). Mean MP was not associated with alveolar CD8 lymphocyte death on day 8. There was no association between mean MP and alveolar NETs on days 1 and 8.

### Correlation of alveolar NETs percentage with lymphocyte/monocyte cell death percentage

Additional file 1: Table S1 in the Supporting Information shows the Pearson’s correlation coefficients between the NETs percentage and other cell death percentages. The percentage of alveolar NETs was not associated with alveolar CD4/CD8 lymphocyte and monocyte cell death percentages on days 1 and 8.

## Discussion

DP and MP were found to be negatively related to cell death in alveolar CD4/CD8 lymphocytes and monocytes. This indicates that the predicted tidal volume can be set without increased DP and MP in patients with higher alveolar lymphocyte and monocyte cell death. The cause might be that the cell death percentages of alveolar CD4/CD8 lymphocytes and monocytes had a tendency to have a positively correlation with dynamic lung compliance. Under the present lung protective strategies, patients with lower dynamic lung compliance require higher DP or MP to maintain a ventilated tidal volume of 6–8 mL/kg PBW.

Ventilated tidal volume was not associated alveolar cell death in this study. This might indicate that the importance of tidal volume was not higher than DP or MP. In the most important study to support low tidal ventilation from the ARDS network [17], stretch-induced lung injury may not occur if lung compliance is not greatly reduced. There was no survival benefit of low tidal volume ventilation in patients with less decreased lung compliance. In patients with severely decreased lung compliance, low tidal volume ventilation could prevent over high DP and MP. Thus, the key factor of VILI might be DP or MP, that had negative relationship with cell death in alveolar CD4/CD8 lymphocytes and monocytes.

We found that the percentages of alveolar CD4/CD8 lymphocyte and monocyte cell death on day 8 were positively correlated with the mean dynamic lung compliance during the eight days. However, there was no correlation between dynamic lung compliance and the percentages of alveolar CD4/CD8 lymphocyte and monocyte cell death on day 1. This indicates that lung compliance was affected by the ventilator setting, such as DP and MP, during these 7 days and finally had a positively correlation with alveolar cell death. This also suggests that high DP or MP might decreased alveolar CD4/CD8 lymphocyte and monocyte cell death through unknown mechanism. ARDS severity is known to be related to the compliance of the respiratory system [1, 22]. Higher lymphocyte/monocyte cell death may result in lower inflammatory immune responses because of a lower percentage of residual functional immune cells with consequence of immunosuppression [23]. A poor inflammatory immune response may prevent further lung injury, resulting in a higher (less decreased) mean dynamic lung compliance.

Apoptosis is the major cause of cell death during infection or inflammation [23]. Steroids are potent regulators inducing apoptosis in steroid-dependent cell types via steroid receptors [24, 25]. Therefore, steroids have been used as induction therapies for pediatric acute lymphoblastic leukemia [26]. In recent reviews, steroid use was encouraged in patients with ARDS [27, 28]. The results of our study support the use of steroids in severe ARDS because increased cell death of alveolar lymphocytes and monocytes is associated with better dynamic lung compliance and decreased disease severity.

In this study, we did not find any association between the percentages of NETs and other types of alveolar cell death on days 1 and 8. NETs degradation attenuates alveolar macrophage pyroptosis in the lipopolysaccharide-induced acute lung injury mouse model [29]. NETs induce alveolar macrophage pyroptosis by activating the nucleotide-binding oligomerization domain leucine-rich repeat and pyrin domain containing 3 (NLRP3) inflammasome, and downregulaton of NETs or targeting components of the NLRP3 inflammasome can effectively attenuate sepsis-induced lung injury [30]. Although NETs appear to increase macrophage death, no study has demonstrated the same effect in lymphocytes. However, the exact effect of NETs on the death of other immune cells requires further investigation.

NETs formation is a common pathogenic mechanism in ARDS [31]. Increased neutrophil apoptosis in ARDS decreases NETs formation, inhibits inflammation, and consequently relieves ARDS [32]. However, this study did not observe an association between NETs percentage and dynamic lung compliance, mean DP, or mean MP. This may be because NETs enhance ARDS development but are not associated with ARDS severity. ARDS severity is associated with the death of alveolar lymphocytes and monocytes.

This study has three major limitations. First, the sample size was not large. However, the number of patients was sufficient and the correlation coefficients were good, which resulted in an ideal estimated statistical power. Second, the tidal volume was not adequately kept at 6–8 mL/kg PBW in this study for many reasons, such as using PCV instead of VCV, hypercapnia, and patient-ventilator unsynchrony even under sedation and paralysis. Although the patients in this study were not ventilated with a low tidal volume, the ventilated tidal volume was approximately 9 mL/kg PBW and not greater than 10 mL/kg PBW. Thus, VILI was less likely to be induced. Third, the static lung compliance was not assessed. Dynamic lung compliance includes chest wall compliance, lung tissue compliance, and airway resistance. Although dynamic lung compliance is not static lung compliance or respiratory system compliance, it can be represented as respiratory system compliance to a certain degree.

## Conclusion

This study showed that patients with higher cell death percentages of alveolar CD4/CD8 lymphocytes and monocytes had higher mean dynamic lung compliance. There was no association between NETs levels and the mean dynamic lung compliance. Patients with decreased alveolar CD4/CD8 lymphocyte and monocyte cell death percentages require increased DP or MP to maintain adequate ventilation. The more severe the ARDS was, the more DP or MP was administered. As our study is the first to present an association between alveolar immune cell death and respiratory mechanics in patients with ARDS, further large-scale studies are required to confirm our results.

### Supplementary Information

**Additional file 1: Table S1.** Pearson correlation between percentages of NETs and other alveolar cell death on Days 1 and 8. **Figure S1.** Flow cytometry of bronchoalveolar lavage (BAL) cells on day 1. Cells in area O were gated as BAL mononuclear cells in the scatterplot of forward scatter (FS) and side scatter (SS) (A). CD4 lymphocytes were identified through positive CD3 and CD4 (B). CD8 lymphocytes were identified through positive CD3 and CD8 (C). Monocytes were identified through positive CD11b and CD14 (D). Different alveolar cell death was detected with positive 7-aminoactinomycin D (7-AAD) and annexin-V (E, F, G, respectively). In this patient, cell death percentages of CD4 lymphocytes, CD8 lymphocytes, and monocytes were 30.97%, 13.92%, and 29.37%, respectively. **Figure S2.** Fluorescence microscopy of alveolar neutrophils. Hoechst 33342 shown in blue and Sytox Green shown in green. Arrows indicate neutrophil extracellular traps (>68 μm^2^). **Figure S3.** Scatterplots showing the Pearson correlation with linear mean regression lines between mean tidal volume and different alveolar cell death on Days 1 and 8. Pearson correlation coefficient (*r*) was calculated. Abbreviation: PBW, predicted body weight; NETs, neutrophil extracellular traps.

## Data Availability

The datasets generated in this study can be obtained on request from the corresponding author.

## Abbreviations

ARDS: Acute respiratory distress syndrome
NETs: Neutrophil extracellular traps
VILI: Ventilator-induced lung injury
DP: Driving pressure
MP: Mechanical power
ICU: Intensive care unit
PCV: Pressure-controlled ventilation
VCV: Volume-controlled ventilation
PBW: Predicted body weight
PSV: Pressure support ventilation
C: Compliance
RR: Respiratory rate
V_T_: Tidal volume
P_insp_: Inspiratory airway pressure
PEEP: Positive end-expiratory pressure
BAL: Bronchoalveolar lavage
7-AAD: 7-Aminoactinomycin D
*r*: Correlation coefficient
NLRP3: Nucleotide-binding oligomerization domain leucine-rich repeat and pyrin domain containing 3
