# Supplementary material for: Cell death of alveolar lymphocytes and monocytes is negatively correlated with driving pressure and mechanical power in patients with acute respiratory distress syndrome
Source: Eur J Med Res. 2024 Jan 3;29:16. doi: 10.1186/s40001-023-01607-4 (PMC10763296; doi:10.1186/s40001-023-01607-4)
Supplement: Supplementary file 1 — Additional file 1: Table S1. Pearson correlation between percentages of NETs and other alveolar cell death on Days 1 and 8. Figure S1. Flow cytometry of bronchoalveolar lavage (BAL) cells on day 1. Cells in area O were gated as BAL mononuclear cells in the scatterplot of forward scatter (FS) and side scatter (SS) (A). CD4 lymphocytes were identified through positive CD3 and CD4 (B). CD8 lymphocytes were identified through positive CD3 and CD8 (C). Monocytes were identified through positive CD11b and CD14 (D). Different alveolar cell death was detected with positive 7-aminoactinomycin D (7-AAD) and annexin-V (E, F, G, respectively). In this patient, cell death percentages of CD4 lymphocytes, CD8 lymphocytes, and monocytes were 30.97%, 13.92%, and 29.37%, respectively. Figure S2. Fluorescence microscopy of alveolar neutrophils. Hoechst 33342 shown in blue and Sytox Green shown in green. Arrows indicate neutrophil extracellular traps (>68 μm2). Figure S3. Scatterplots showing the Pearson correlation with linear mean regression lines between mean tidal volume and different alveolar cell death on Days 1 and 8. Pearson correlation coefficient (r) was calculated. Abbreviation: PBW, predicted body weight; NETs, neutrophil extracellular traps. [file 40001_2023_1607_MOESM1_ESM.docx]

Table S1. Pearson correlation between percentages of NETs and other alveolar cell death on Days 1 and 8

|  | Pearson’s *r* | *p* value |
| --- | --- | --- |
| Day 1 | | |
| CD4 lymphocytes | 0.194 | 0.363 |
| CD8 lymphocytes | 0.208 | 0.330 |
| Monocytes | 0.296 | 0.160 |
| Day 8 | | |
| CD4 lymphocytes | -0.087 | 0.686 |
| CD8 lymphocytes | 0.263 | 0.214 |
| Monocytes | 0.060 | 0.780 |

Abbreviation: NETs, neutrophil extracellular traps; *r*, correlation coefficient


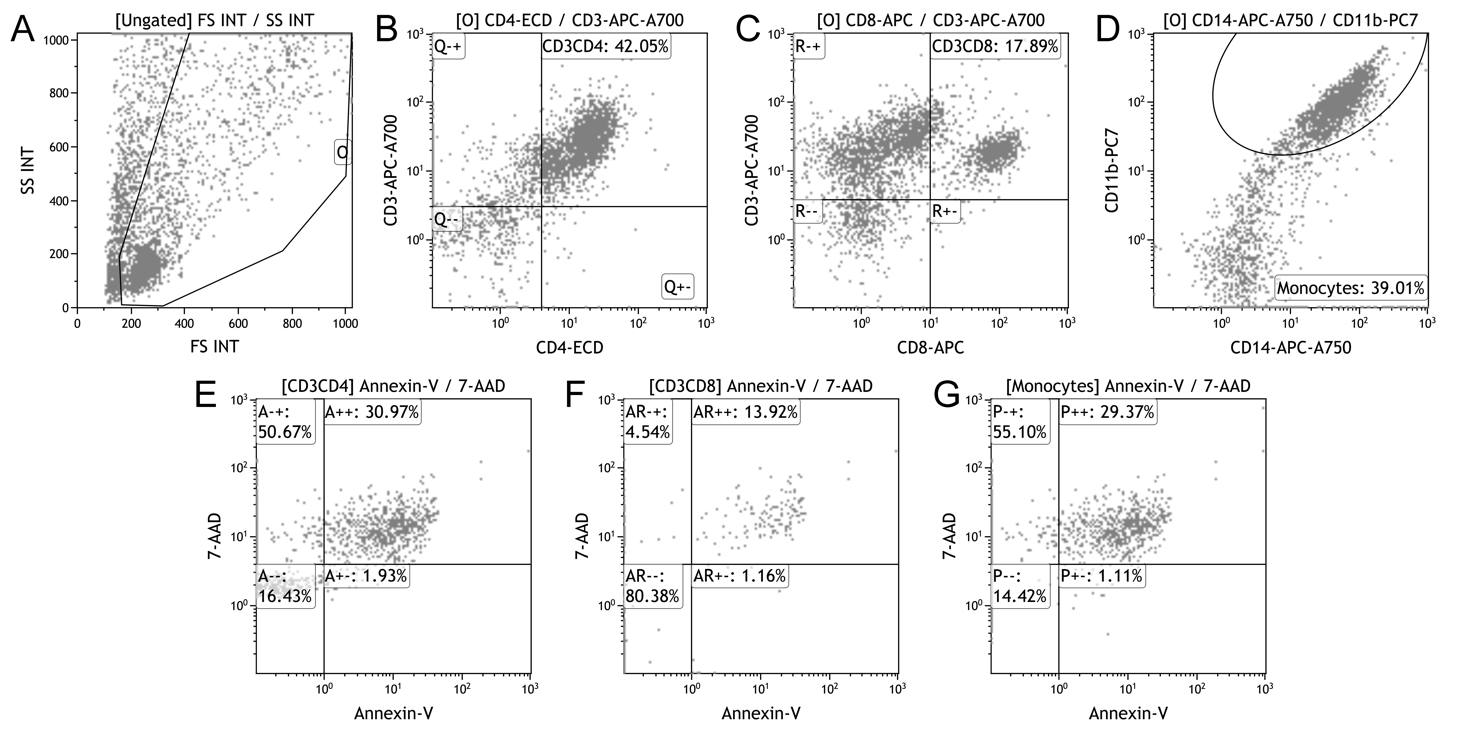


Figure S1. Flow cytometry of bronchoalveolar lavage (BAL) cells on day 1. Cells in area O were gated as BAL mononuclear cells in the scatterplot of forward scatter (FS) and side scatter (SS) (A). CD4 lymphocytes were identified through positive CD3 and CD4 (B). CD8 lymphocytes were identified through positive CD3 and CD8 (C). Monocytes were identified through positive CD11b and CD14 (D). Different alveolar cell death was detected with positive 7-aminoactinomycin D (7-AAD) and annexin-V (E, F, G, respectively). In this patient, cell death percentages of CD4 lymphocytes, CD8 lymphocytes, and monocytes were 30.97%, 13.92%, and 29.37%, respectively.


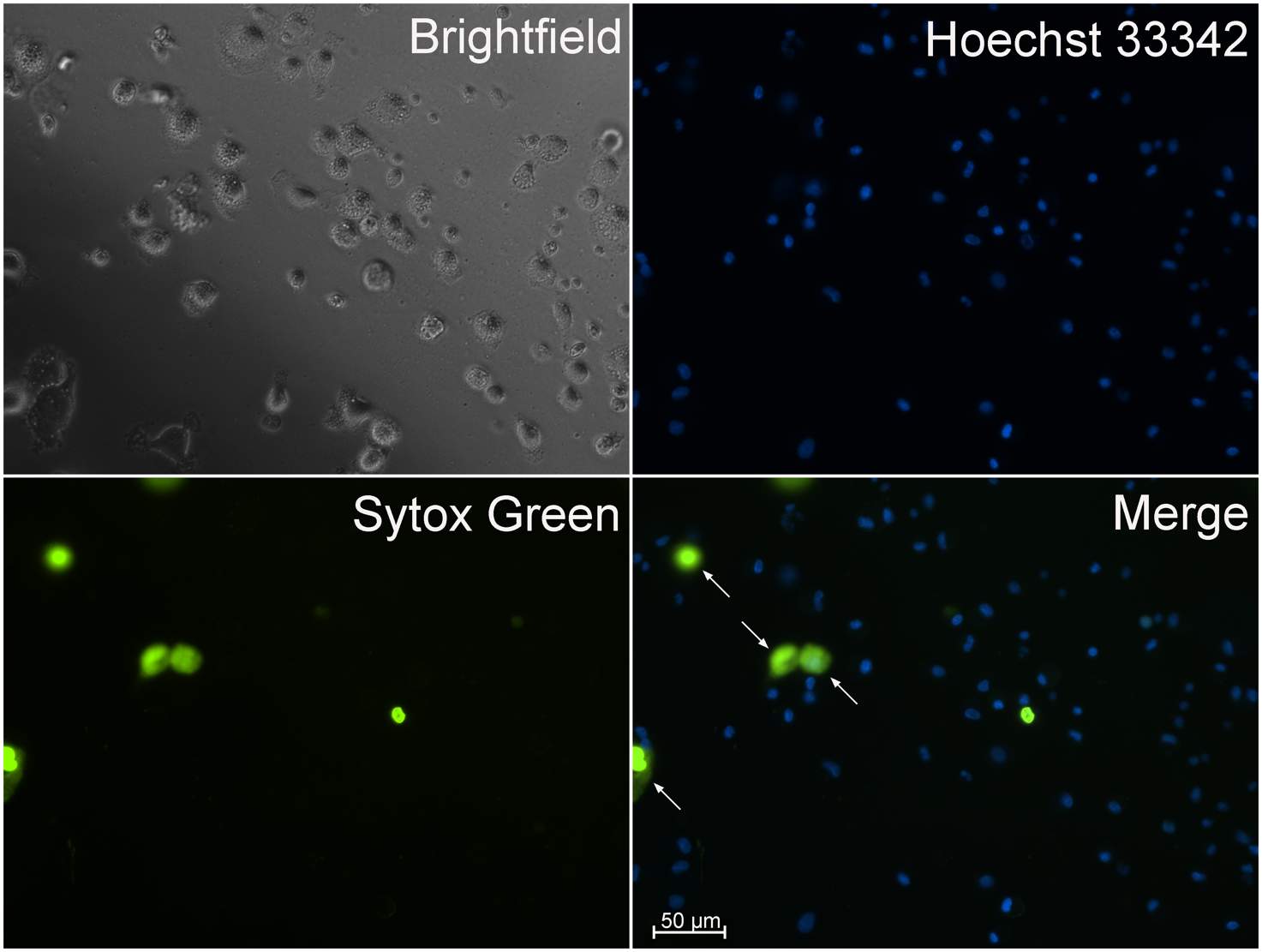


Figure S2. Fluorescence microscopy of alveolar neutrophils. Hoechst 33342 shown in blue and Sytox Green shown in green. Arrows indicate neutrophil extracellular traps (>68 μm^2^).





Figure S3. Scatterplots showing the Pearson correlation with linear mean regression lines between mean tidal volume and different alveolar cell death on Days 1 and 8. Pearson correlation coefficient (*r*) was calculated. Abbreviation: PBW, predicted body weight; NETs, neutrophil extracellular traps.
